# Partial sterno-costo-claviculectomy for Ewing’s sarcoma of the medial clavicle (case report)

**DOI:** 10.1016/j.ijscr.2023.109213

**Published:** 2024-01-03

**Authors:** A. Rajaallah, F. Lamnaouar, A. Rafaoui, A. Messoudi, M. Rahmi, M. Rafai

**Affiliations:** a Orthopedics and Traumatology Surgery Professor at the Pavilion 32 CHU Ibn Rochd of Casablanca, Morocco; b Resident in Orthopedics and Traumatology Surgery at the Pavilion 32 CHU Ibn Rochd of Casablanca, Morocco

**Keywords:** Ewing's sarcoma, Clavicle, claviculectomy, primary tumor, biomechanical function

## Abstract

**Introduction:**

Primary clavicle tumors are uncommon and account for <1 % of primary bone tumors. The majority of primary clavicle tumors are malignant. Approximately 1.4 % of all cases of Ewing's sarcoma occur in the clavicle.

**Case presentation:**

Here, we report the case of a patient diagnosed with Ewing's sarcoma who received neoadjuvant chemotherapy and had an important reduction in the tumoral volume; second, the patient had undergone partial resection of the medial clavicle, first rib, and the sternum.

**Discussion:**

The survival rate of patients with nonmetastatic lesions is 56–79 %. The clavicle is connected to major anatomical structures, which makes surgery challenging. Even if there is little morbidity after resection, these studies did not show the superiority of reconstruction.

**Conclusion:**

The location of Ewing's sarcoma in our patient indicated that the surgical approach was needed for large resection. The functional outcomes were excellent, with no signs of recurrence at the 2-year follow-up.

## Highlights

• Primary clavicle tumors are uncommon and account for <1% of primary bone tumor.1.4% of all cases of Ewing's sarcoma occur in the clavicle.
• adequate resection (partial or total claviclectomy) is the treatment of choice for local tumor control
• The clavicle has close links with major anatomical structures, and its approach is a tough challenge, particularly in the case of a large tumor mass
• Morbidity of the upper limb from a claviculectomy is obviously a concern. Reconstructive surgery could expose the patient to the risk of surgery, although there is no functional superiority
• reported a survival rate of 72% for peripheral lesions and 78% for non-metastatic lesions

## 1 Introduction

Primary tumors of the clavicle are uncommon, accounting for <1 % of primary bone tumors [1], and are most malignant [2]. In the absence of secondary locations, treatment remains conservative [3].

Ewing's sarcoma occurs in the clavicle in 1.4 % of cases [4]. Tumor resection remains the preferred modality for local tumor control, and chemotherapy is still used in combination with radiotherapy.

We report the case of a patient with chemosensitive Ewing's sarcoma of the right clavicle who underwent radical treatment consisting of partial costo-sterno-claviculectomy in addition to chemotherapy.

## 2 Observation

The reporting of this work follows the SCARE checklist criteria [14], ensuring adherence to guidelines for quality reporting in case series.

Mrs. A.R., aged >20, right-handed, with no previous history of any particular complaint, presented to the clinic with painful swelling that had developed for 2 years on the right clavicle and had progressively increased in size in the context of weight loss that had not been quantified and apyrexia.

**Fig. 1:**
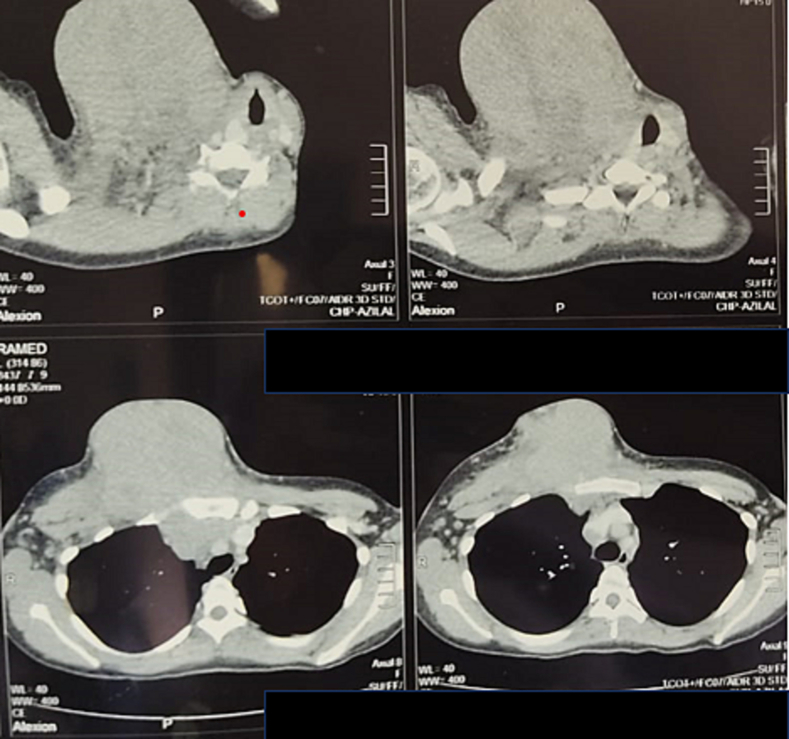
Chest CT scan before chemotherapy showing a large tissular tumor of 15 cm along its long axis in front of the right medial clavicle.

The patient underwent a standard radiological work-up with an X-ray of the shoulder and a CT scan, which showed the presence of a tumor process developing at the base of the neck and in the anterior part of the thoracic cavity, with invasion of the anterior mediastinum (Fig. 1).

The patient underwent a biopsy, the results of which showed the presence of a neuroectodermal tumor (Ewing's sarcoma), after which it was decided to undergo neoadjuvant chemotherapy before surgery. The patient underwent 10 sessions of neoadjuvant chemotherapy (VAC IE protocol), and there was a marked regression in the volume of the mass.

**Fig. 2:**
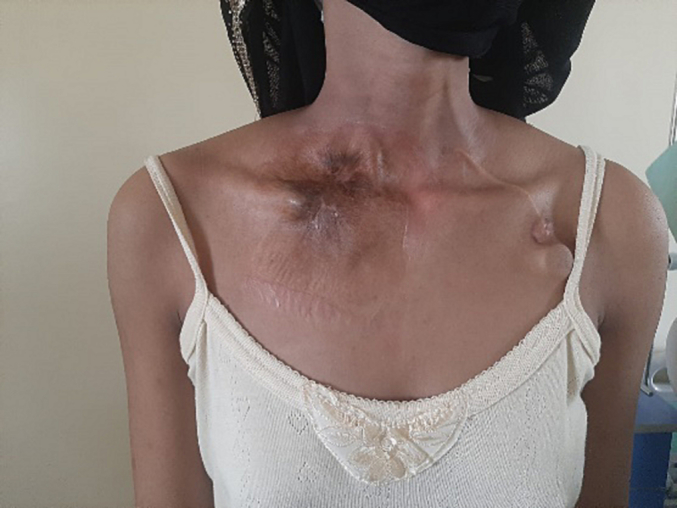
Scar appearance of the skin on the right, the implantable chamber on the left.

Physical examination on admission revealed a patch of scarring and discoloration of the skin over the medial half of the right clavicle, with no vascular-nervous disorders of the right upper limb (Fig. 2).

**Fig. 3:**
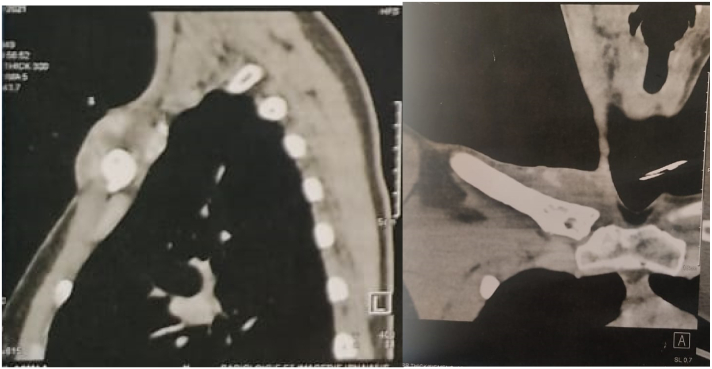
Post-chemotherapy CT scan showing type condensation of the clavicle and sternal manubrium.

**Fig. 4:**
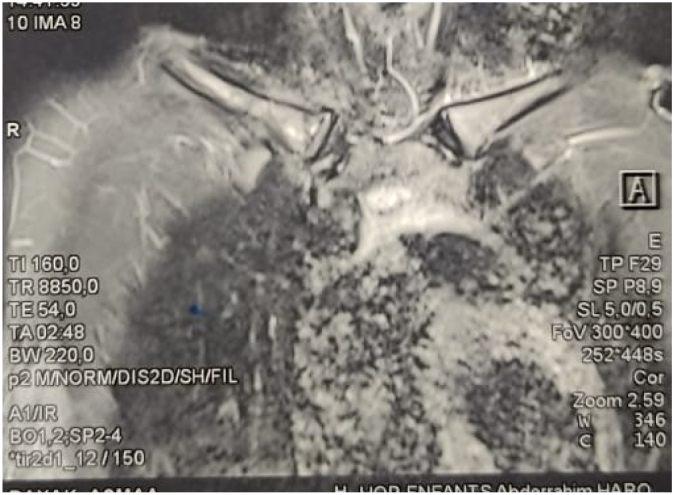
MRI image of the sternal end of the clavicle showing an abnormal signal from the sternal manubrium.

A chest X-ray showed a mitted appearance at the proximal end of the right clavicle. The follow-up CT scan (Fig. 3) and MRI (Fig. 4) showed almost complete regression of the tumor mass, with a hyper-condensed appearance at the proximal end of the clavicle and the sternal manubrium (Fig. 3).

**Fig. 5:**
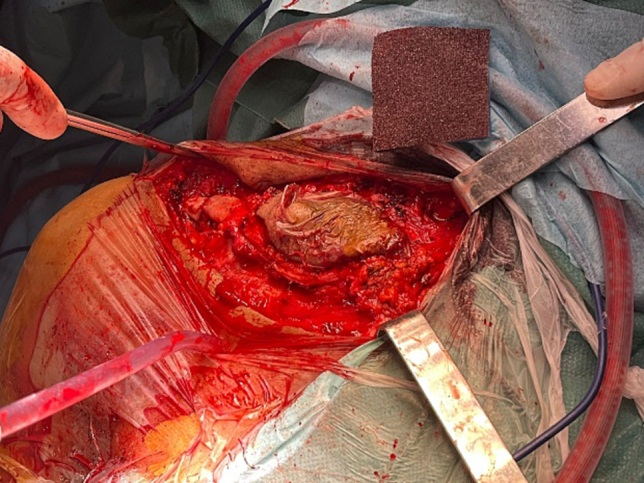
Approach for resection of orange-peel skin.

**Fig. 6:**
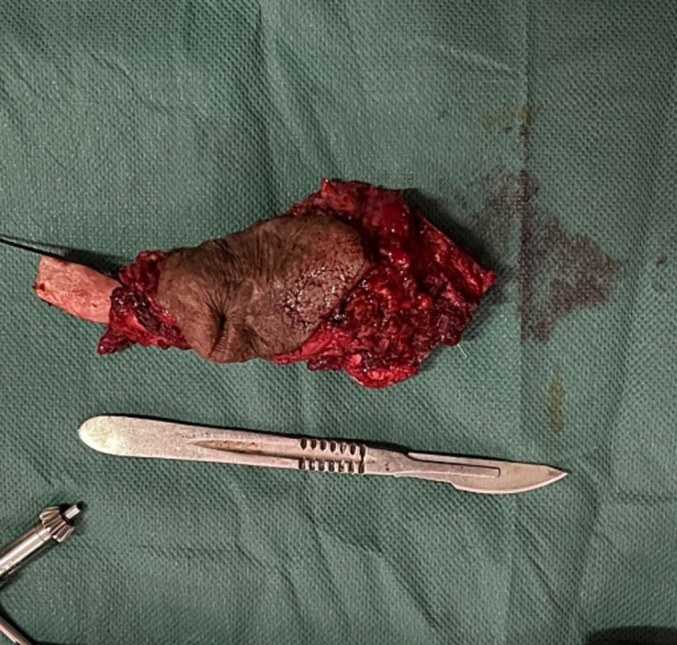
Resection piece for partial sterno-cleido-costectomy with resection of the surrounding skin.

The patient underwent a monobloc resection of the clavicle, the subclavius muscle, the first rib, and part of the sternal manubrium (Fig. 5, Fig. 6). surgery which was made by a team of Orthopedic and thoracic surgery, the beginning was by a skin incision of orange-peel around the scarring part, dissection realized to locate and protect the subclavicular pedicle, and a monobloc resection of the clavicle the anterior part of the first rib, the subclavius muscle and a part of the sternum was taken after an osteotomy with electric saw.

**Fig. 7:**
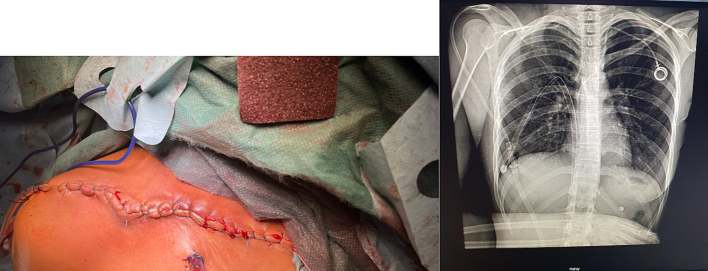
Control X-ray taken on day 1 postop with the chest drain still in place.

A thoracic drain was inserted. No complications in the postoperative period with well-controlled analgesia (Fig. 7).

The patient was seen via consultation 3 weeks, 6 weeks, and 3 months after the procedure and every 6 months for 2 years after surgery. The scar was clean and free of any signs of inflammation.

**Fig. 8:**
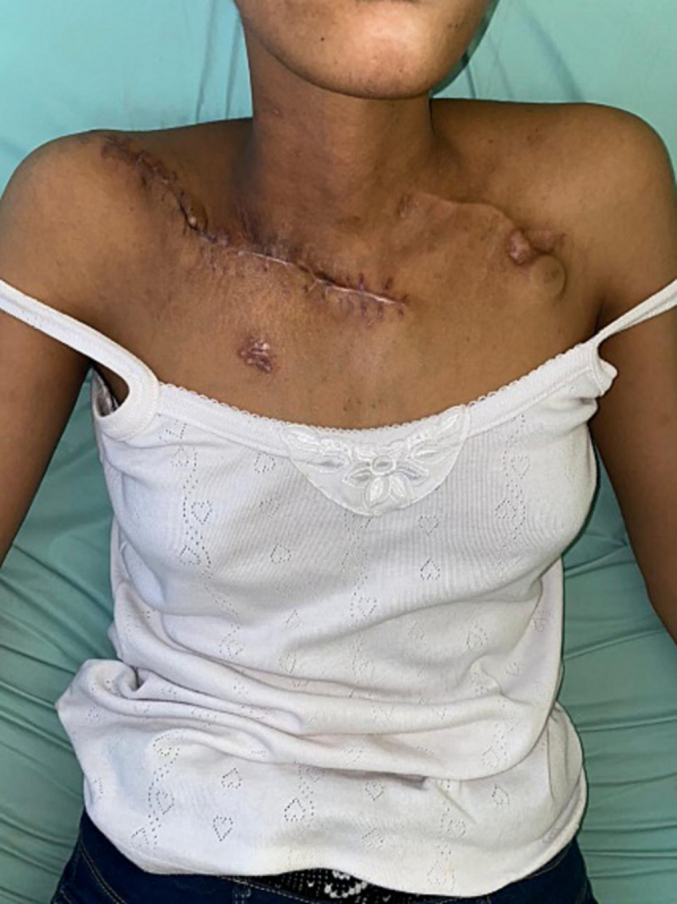
The surgical scar at 2 months.

**Figs. 9, 10:**
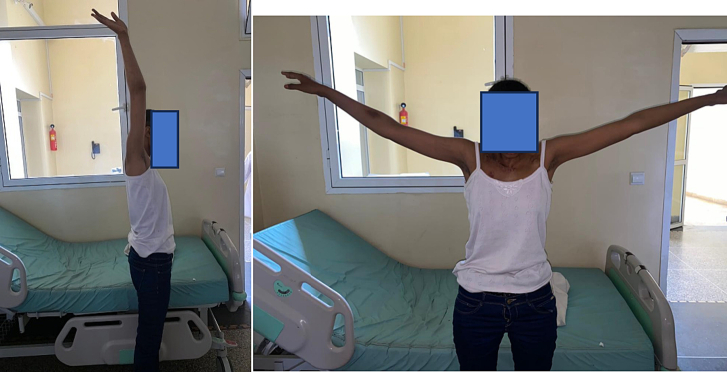
Examination of active joint range in the right shoulder.

Examination of active joint amplitudes revealed functional recovery with an abduction movement of up to 100, an anteflexion movement of 180°, and an extension of 80° (Figs. 8, 9, 10).

## 3 Discussion

Primary tumors of the clavicle are rare, accounting for <1 % of bone tumors [1]. In a study by Klein, tumors of the clavicle accounted for 0.45 % of 13,000 primary bone tumors, with a predominance of the acromial extremity [5].

Ewing's sarcoma is a primary malignant tumor that originates in the bone marrow. Known for its high metastatic potential, the prognosis of Ewing's sarcoma is even worse in the clavicular location, given the vascular invasion it causes and the therapeutic difficulty [6].

As with other primary tumors, adequate resection (partial or total claviculectomy) is the treatment of choice for local tumor control. However, multidisciplinary management is required to treat this tumor appropriately [7].

The clavicle is closely related to major anatomical structures, and accessing this space is a difficult challenge, particularly for large tumor masses [1].

**Fig. 11:**
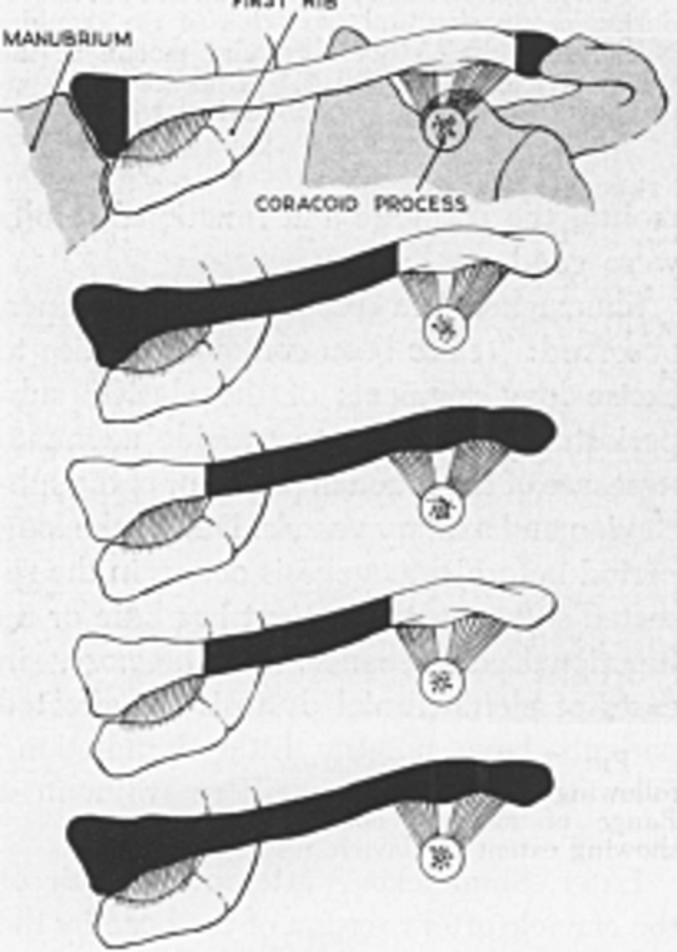
[Abbott et al] Theoretical sites for partial and complete resection of the clavicle. The dark areas indicate the regions to be resected [8].

The clavicle has four functions: acting as a prop. The second function constitutes a framework for muscle origins and insertions. Third, it provides bony protection for the subclavian and axillary plexus. The fourth function, which is the most important, is to provide a means of transmitting the supporting force of the trapezius to the scapula through the coracoclavicular ligament. Resection should be carried out to conserve the function of the shoulder girdle and avoid pain or deformity [8]. Abbott et al. established a list of theoretical sites for resection to avoid, at best, biomechanical complications (Fig. 11).

Morbidity of the upper limb after claviculectomy is a concern. There are different opinions as to whether reconstruction is necessary after excision, and studies have reported shoulder dysfunction and scapular dyskinesia [7]. Reconstructive surgery could expose the patient to the risk of surgery, although there is no functional superiority [9].

The prognosis varies in severity, depending on the age of onset, the presence or absence of metastases, and the presence or absence of a response to chemotherapy [10]. Pradhan et al. [11] reported a survival rate of 72 % for peripheral lesions, 78 % for nonmetastatic lesions, and 56–79 % in a study by Nesbit et al. [12].

## 4 Conclusion

Clavicle counts less than 1 % of primary bone tumor localization, the majority of those tumors are malignant. Tumor resection remains the best therapeutic choice.

Partial or total resection of the clavicle according to the different reviews did not affect the function of the upper member.

## Consent

Written informed consent was obtained from the patient for publication of this case report and accompanying images. A copy of the written consent is available for review by the Editor-in-Chief of this journal on request.

## Ethical approval

The study is exempt from ethnical approval in our institution for being just a case report only the patient consent is necessary (comité d'ethique du centre hospitalier IBN Rochd de Casablanca).

## Funding

This research received no specific grant from funding agencies in the public, commercial, or not-for-profit sectors.

## Author contribution

Dr Rajaallah Abdssamad: the operator, reviewed the paper

Dr Foad Lamnaouar, writing the paper

Dr Abderahim Rafaoui reviewed the paper

Dr Abdeljebbar Messoudi reviewed the paper

Dr Rahmi Mohamed reviewed the paper

Dr Rafai Mohamed: the operator reviewed the paper/concept

## Guarantor

Pr Rafai Mohamed.

## Declaration of competing interest

The authors declare no conflicts of interest.
